# Staphylococcal superantigen-specific IgE antibodies and its correlation with asthma severity

**DOI:** 10.1186/1939-4551-8-S1-A44

**Published:** 2015-04-08

**Authors:** José Elabras Filho, Alfeu Tavares França, José Angelo De Souza Papi, Blanca Elena Rios Gomes Bica, Omar Lupi

**Affiliations:** 1 Hospital Universitário Clementino Fraga Filho Hucff-Ufrj, Brazil; 2 Hospital São Zacharias, Brazil

## Background

Elevated IgE antibodies to Staphylococcal toxins (superantigens) are related to asthma severity in a few studies. We have investigated this hypothesis in an adult asthmatic population from the Clinical Immunology outpatient service of an University Hospital. Our objectives were: to detect the presence and the degree of IgE-mediated sensitization to staphylococcal toxins *in vitro* in asthma patients; to correlate the presence and concentration of specific IgE against staphylococcal toxins with asthma severity; and to assess whether elevated levels of serum IgE specific for staphylococcal toxins may have predictive value for asthma severity.

## Patients and methods

We studied 142 patients, diagnosed by clinical spirometric findings as asthmatics, attended at the Clinical Immunology outpatient service of an University Hospital. They were divided into two groups according to their asthma severity index: group 1 (n=72), mild intermittent or mild persistent asthma, and group 2 (n=70) with moderate or severe asthma. After clinical history and physical examination, they were screened for serum specific IgE against staphylococcal enterotoxins types A (TXA), B (TXB), C (TXC) and TSST (toxic shock syndrome toxin).

## Results

Patients mean age was 49.5 years-old, most of them were female and caucasian. 26.1% of them were former smokers. The majority also had rhinitis, positive prick tests for inhalant allergens, and family history of atopy. 62 patients had a positive IgE dosage (43,7%): 29 for TXA (20,4%), 35 for TXB (24,6%), 33 for TXC (23,2%), e 45 for TSST (31,7%). The mean dosage of the positive tests were: TXA - 0,96 U/L, TXB - 1,09 U/L, TXC - 1,21, TSST - 1,18 U/L. There were no significant differences between the number of positive tests or mean dosage when compared both groups 1 and 2.

## Conclusions

In our study, IgE anti-staphylococcal TXA, TXB, TXC and TSST were not significant related to asthma severity, and had no predictive value in relation to asthma severity.

